# Protein Counting as an Educational Strategy to Optimize Low-Protein-Diet Adherence and Satisfaction in Stage 4 and 5 Chronic Kidney Disease Patients: A Pilot Study

**DOI:** 10.3390/nu17091438

**Published:** 2025-04-25

**Authors:** Francesca K. Martino, Lucia F. Stefanelli, Alessandra Zattarin, Larissa Lovato Correa Dias, Greta Redi, Rime Khalf, Dorella Del Prete, Federico Nalesso

**Affiliations:** 1 Nephrology, Dialysis, Transplantation Unit, Department of Medicine (DIMED), University of Padova, 35128 Padua, Italy; luciafederica.stefanelli@unipd.it (L.F.S.); rime.khalf@studenti.unipd.it (R.K.);; 2 Department of Medicine (DIMED), Clinical Nutrition, University of Padua, 35128 Padua, Italy; alessandra.zattarin@aopd.veneto.it (A.Z.); lcdlarissa@gmail.com (L.L.C.D.); greta.redi@studenti.unipd.it (G.R.)

**Keywords:** low-protein diet, protein counting, chronic kidney disease, adherence, satisfaction

## Abstract

Background/Objectives: Educational support is a well-established strategy in diet management for chronic diseases. No study has reported the impact of protein counting in a low-protein diet for chronic kidney disease (CKD). We aimed to assess how a protein-counting educational program could enhance adherence and satisfaction in managing a low-protein diet in CKD patients with an eGFR below 20 mL/min/1.73 m^2^. Methods: We conducted a prospective pilot study comparing adherence and satisfaction before and after an educational program, which included four group meetings and two individual meetings over six months. Diet adherence was assessed by estimating protein intake using 24 h urine urea excretion, while diet satisfaction was evaluated with the MDRD questionnaire. Results: Twenty-four patients with a mean age of 63.9 years experienced improved diet adherence, with a significant reduction in protein intake (from 59.82 ± 15.57 g/day to 47.18 ± 13.7 g/day, *p* < 0.001) and a significant increase in overall diet satisfaction (from a median of 3.57 (3.1–4) to 4 (3.6–4.1), *p* = 0.001). Conclusions: An educational program that includes protein counting assists CKD stage 4–5 patients in improving their diet adherence and increasing overall dietary satisfaction.

## 1. Introduction

Chronic kidney disease (CKD) is a global health concern, affecting approximately 11–13% of the adult population [1,2,3] and over 25% of older adults [3,4,5,6]. Conservative management (CM) is a key strategy that combines lifestyle interventions, pharmacological therapy, and dietary–nutritional therapy to slow down the progression of the disease, delaying the need for dialysis or kidney transplantation in patients with end-stage kidney disease (ESKD) [7,8,9]. CM requires a multidisciplinary approach in which personalized dietary interventions with a low-protein diet play a crucial role [7]. Specifically, a low-protein diet helps maintain an effective electrolyte–acid balance. It reduces uremic toxin levels even in the advanced stages of CKD without increasing the risk of sarcopenia [10] and contributing to an improvement in quality of life [11,12]. Protein restriction is usually prescribed based on residual kidney function, and at CKD stages 4 and 5, it can reach 0.3–0.4 g/kg/day with Ketoanalogs support [7]. Adhering to a low- or very-low-protein diet can be challenging for CKD patients [13], involving a restriction in protein foods and consistent dietary effort [14]. However, optimizing adherence improves prognosis regarding mortality and morbidity in CKD patients [15,16,17] and should be pursued in every possible way.

In CKD management, educational interventions showed improved adherence to a low-phosphate diet in CKD [18,19] and to a low-protein diet [20]. Enhancing knowledge about protein in food and the self-management of dietary protein restrictions could be a suitable strategy to improve diet adherence in CKD patients [21,22,23], allowing a wider dietary variety and less restrictive meals. Thus, educational interventions could improve awareness about CKD management and improve quality of life. The educational approach has already shown its value in diabetes [24,25,26], where managing carbohydrate intake through carbohydrate-counting techniques led to greater adherence to therapy and improved health-related quality of life in diabetes patients. In general, carbohydrate and, hopefully, protein counting can allow one to manage meals more flexibly and positively impact the social sphere and overall quality of life [27,28,29,30,31].

From this perspective, our pilot study serves as the first step in evaluating the role of protein counting on diet adherence in CKD patients and the possible benefits of this approach in managing low- and very-low-protein diets. Our educational approach consisted of small-group meetings with dieticians, who focused on explaining low-protein-diet efficacy in slowing CKD progression and the provision of practical tools for protein counting to optimize diet adherence. The lack of previous studies about protein counting in CKD settings [32] required us to perform a preliminary evaluation to assess the possible procedure flaws and to conduct a conclusive finding in the future.

## 2. Materials and Methods

We conducted a prospective study on a cohort affected by CKD who were undergoing conservative management at the University Hospital of Padua.

We considered all patients with CKD stages 4 and 5 eligible for the trial, who followed a low-protein diet for at least six months, and freely accepted to join this study. Exclusion criteria were a lack of informed consent to participate in this study and the presence of any impairment that could preclude an adequate adherence to a low-protein diet, such as the following:

- Pregnancy;
- Cognitive impairment or learning impairment;
- Alcohol or drug abuse;
- Psychiatric disease;
- Gastric and bowel enteric disease (inflammatory bowel disease, celiac disease, Whipple’s disease, and enterostomy).

### 2.1. Educational Program

**Figure 1:**
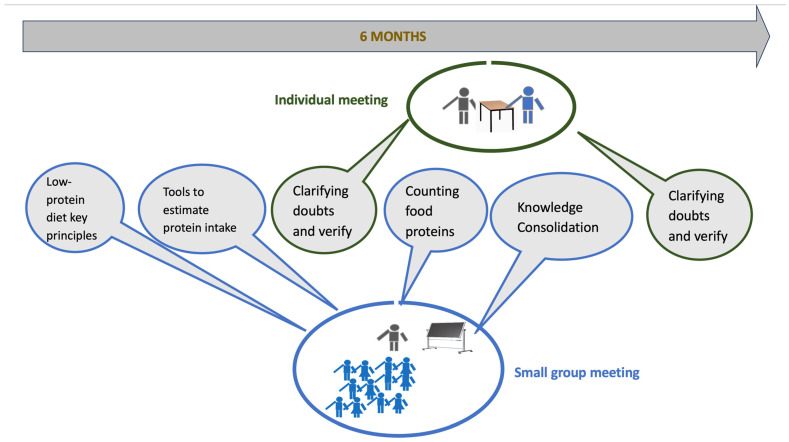
Six-month educational program.

The participants underwent an educational intervention consisting of four group sessions and two individual sessions led by a dietitian, distributed over six months, as reported in Figure 1:

- The first meeting illustrated the renal functions and the rationale for low-protein diets. Specifically, we focused on the benefit of kidney function with a low-protein diet, protein needs, vegetable and animal protein sources, and other diet changes with a protein diet.
- The second meeting guided patients in using tools for a low-protein diet, such as reading food labels, estimating portions, and cooking tricks.
- The third meeting was entirely dedicated to counting the proteins contained in foods.
- The fourth meeting focused on practical exercises aimed at consolidating the learned notions, recipes, and practical advice for the effective management of the low-protein diet.
- The two individual sessions aimed to clarify doubts and verify the patients’ correct counting application.

### 2.2. Clinical Evaluation

Each patient underwent a thorough clinical evaluation at two key time points: T0 (upon enrollment) and T1 (after the educational program).

- Epidemiological parameters evaluated: age, gender, and medical comorbidities such as diabetes, hypertension, heart failure, liver disease, and chronic obstructive pulmonary disease (COPD).
- The anthropometric data included weight, height, and body mass index (BMI).
- Biochemical parameters included creatinine, plasma urea, uric acid, serum sodium, potassium, calcium, phosphorus, albumin, hemoglobin, blood glucose, 24 h of proteinuria, and 24 h of urine excretion of urea and sodium.
- In cooperation with the patient, we reviewed a three-day dietary diary completed at home before the visit. The data on nutrient intakes (calories, proteins, carbohydrates, lipids, fibers, sodium, phosphorus, and potassium) were obtained using the Italian food composition tables [32]. The intakes were calculated using the Metadieta software (Meteda—METEDA S.r.l. San Benedetto del Tronto (AP), Italy).
- The DSQ-MDRD questionnaire to assess dietary satisfaction was completed (Appendix A). Specifically, we administered the Italian version of the DSQ-MDRD questionnaire, which has 18 items and 5 domains. Every item was graded from 1 to 5, where 1 represents total dissatisfaction and 5 represents complete satisfaction [33]. The five domains represent general satisfaction, ease in food preparation, social difficulties in following the diet, personal perception of diet adherence, and the level of motivation to follow the diet. The mean of each domain was calculated for each patient.

### 2.3. Primary Endpoint

We aimed to assess diet adherence by estimating protein intake. Specifically, dietary protein intake was determined at baseline from urea excretion in 24 h urine by the Maroni equation:

Dietary protein intake (g) = 6.25 × ([urinary urea excretion (g/24 h)] + [0.031 × body weight (kg)]) + urinary protein excretion (g/24 h) [34,35].

Furthermore, to strengthen the evaluation of protein intake, we adopted a three-day dietary diary completed at home.

### 2.4. Secondary Endpoint

Finally, we aimed to assess the impact of the educational program on dietary satisfaction, kidney failure progression, and malnutrition appearance. The first feature was determined according to the MDRD questionnaire, which we considered adequate, with an improvement of >20% in the MDRD questionnaire scores. The stability of kidney function was assessed by creatinine clearance, which we considered stable when creatinine clearance remained at ±10% compared to baseline. Malnutrition was assessed by unchanged BMI (±10% compared to baseline), albumin, and pre-albumin levels within the laboratory normal range at T1.

### 2.5. Statistical Analysis

Categorical variables were presented as percentages and absolute numbers. Depending on their distribution, numeric variables were reported using either the mean and standard deviation or the median and standard deviation. Comparisons between measurements at T0 and T1 were assessed using paired *t*-tests for normally distributed variables or the Wilcoxon signed-rank test for non-normally distributed variables. Depending on their distribution, correlations among numerical variables were evaluated using Pearson or Spearman correlation coefficients.

Differences were considered significant at *p* ≤ 0.05 on both sides.

The analyses were conducted using SPSS software version 28.

### 2.6. Sample Size

We conducted a pilot study to assess protein counting as an educational strategy to optimize low-protein-diet adherence. According to the Hetzog report [36], we considered a suitable sample size between 20 and 40 cases. Specifically, we enrolled 24 patients. Based on our clinic’s patient volume, we established an enrollment period of one month to avoid possible selection bias, during which all patients who accepted to join the study were freely enrolled.

Finally, we estimated the sample size of future studies using the OpenEpi calculator (https://www.openepi.com/SampleSize/SSMean.htm, accessed on 10 January 2025) to compare two means with a power of 80%.

### 2.7. Feasibility Criteria

We considered the current study design fair in feasibility when at least 90% of enrolled patients completed all procedures, performing the preliminary and subsequent tests. Furthermore, we considered all the possible procedural faults to overcome a similar problem in future studies.

## 3. Results

### 3.1. Pilot Study Finding

This study included 26 participants, with a gender distribution of 75% men and 25% women. The mean age of the population was 63.9 years ± 13.5. In total, 62.5% of the participants had a high school diploma, 29.2% had a university degree, and 8.3% had only completed middle school. All patients followed a low-protein diet. Among the associated comorbidities, 91.7% were affected by hypertension, 29.2% by diabetes mellitus, 4.2% by ischemic heart disease, 8.3% by cardiac arrhythmia, 12.5% by chronic obstructive pulmonary disease (COPD), and 8.3% by chronic liver disease.

After the educational program, we identified a significant improvement of biomarkers related to the metabolic control of CKD, with serum bicarbonate levels increasing from 23.26 ± 2.45 mmol/L to 24.16 ± 2.99 mmol/L (*p* = 0.006), plasma urea decreasing from a median value of 18.80 (12.9–23.6) mmol/L to 14.39 mmol/L (10.9–20.1 mmol/L), (*p* < 0.001), and 24 h urinary urea decreasing from 0.26 (0.19–0.32) g/24 h to 0.19 (0.15–0.23) g/24 h (*p* < 0.001). Furthermore, we identified a significant statistical reduction in serum albumin (from 43.58 ± 3.78 g/L to 41.71 ± 3.69 g/L, *p* = 0.005) and in cholesterol values (from 4.60 ± 1.28 mmol/L to 4.14 ± 1.13 mmol/L, *p* = 0.033). The results of the blood chemistry tests are reported in Table 1.

Interestingly, from the analysis of the three-day diet diary before and after the intervention, carbohydrate, lipid, and fiber intake significantly increased. At the same time, considering the three-day diet diary, the average protein phosphate consumption did not reach a statistically significant decrease, as reported in Table 2.

**Figure 2:**
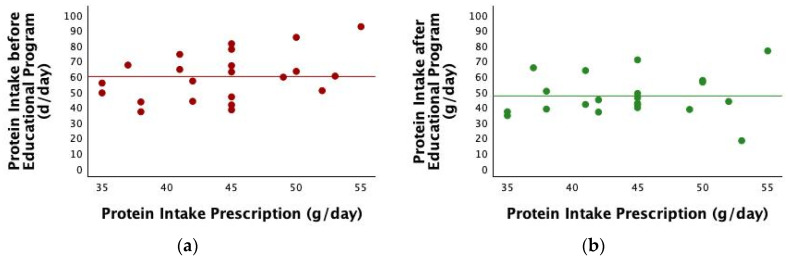
(**a**) Scatterplot of protein intake before the educational program estimated by the Maroni formula according to protein prescription. (**b**) Scatterplot of protein intake after the educational program estimated by the Maroni formula.

However, diet adherence after the educational program significantly improved with an estimation of protein intake by the Maroni formula from 59.82 ± 15.57 g/day to 47.18 ± 13.7 g/day (*p* < 0.001). Figure 2 reports the estimations before and after the educational program according to the prescribed protein intake.

The MDRD questionnaire demonstrated an increase in satisfaction levels across all categories. Table 3 presents the results for the five domains: general satisfaction, ease in food preparation, social challenges in adhering to the diet, personal perceptions of diet adherence, and motivation to follow the diet.

### 3.2. Evaluation of Future Study

According to the mean difference in protein intake evaluated by the Maroni equation (59.82 ± 15.57 g/day before the educational program and 47.18 ± 13.7 g/day after the educational program), the sample size was calculated to be 22 subjects. When considering the difference in protein intake evaluated by the three-day diet diary (45.6 ± 9.7 g/day before the educational program and 42.3 ± 7.1 g/day after the educational program), the sample size increased to 102 patients.

All 24 patients who freely agreed to participate in this study completed all the required procedures, including the six dietician meetings and all of the questionnaires. Two patients refused to join this study because of the overlap with work commitments, and one refused to join because of a lack of interest. Thus, about 11% (3/27) of the possible candidates did not join the study.

## 4. Discussion

The educational support of protein counting enhances adherence to low-protein diets in CKD patients. This improvement aids in metabolic control by reducing urea and phosphate retention, increasing overall diet satisfaction, and bolstering awareness and motivation while mitigating malnutrition risk and glucose metabolism impairment.

### 4.1. Pilot Study Results

Our preliminary findings confirmed the findings of previous studies that highlighted the importance of educational support in enhancing adherence to a restrictive diet for chronic diseases. In patients with diabetes, improved knowledge and skills for self-monitoring and self-managing glycemic control were linked to better metabolic conditions [37,38,39]. Additionally, in hypertension, the long-term modest reduction in salt achieved through educational campaigns [40,41,42] improved blood pressure control [43] and significantly lowered cardiovascular risk [44,45]. The educational focus on nutritional knowledge also improved diet adherence in CKD [46,47] and dialysis [46,48,49] patients. Currently, no studies have assessed protein counting in CKD patients within the context of a low-protein diet. Our pilot study is the first report to determine the beneficial role of protein counting in a low-protein diet. Our results showed that giving CKD patients adequate tools to manage protein intake effectively improves adherence to a low-protein diet and the perception of their quality of life, according to the significant increase in MDRD questionnaire results. Eating is a physiological need that significantly impacts social activities, the patient’s perception of quality of life, and the motivation to follow the dietary plan. CKD patients who follow a low-protein diet, and other patients with diabetes or celiac disease with a restrictive diet, face difficulties in eating in restaurants or having a meal with others, which negatively impacts the patient’s social sphere and worsens their attitude toward following the diet [50,51]. Our study moves in the same direction as previous reports on diabetes or celiac disease, which showed a beneficial effect of educational support [52,53]. Moreover, it emphasizes the importance of the social difficulties of CKD patients in a clinical setting. Chronic patients could benefit from group events because they could reduce isolation by sharing personal experiences, even beyond improving protein intake knowledge. This aspect could be related to the significant increase in general satisfaction and improved social difficulties, adherence perception, and motivation.

Despite the encouraging results, this pilot study necessitates further research to confirm our findings, considering that we did not apply multiple *p*-value corrections to maximize the covariate value in a single comparison. However, based on the Maroni formula for estimating protein intake, the sample size evaluation revealed an extremely low number of patients, reducing concerns about the reliability of our findings. Despite the lack of significant difference between the estimated protein intake by the three-day diet diary, we are confident that we can corroborate our preliminary findings in a future study. As every nephrologist knows in different fields, a biomarker can have different usefulness and meaning according to the target population and the conditions of use [54,55,56,57]. In this specific field, the estimation of protein intake using the 24 h urinary urea excretion represents a more objective estimation than a three-day diet diary. Still, it is based on some assumptions, such as the absence of anabolic or catabolic states and the stability of hydration status [34]. Some of these assumptions could falter in stage 4 and 5 CKD patients, making protein intake estimation less reliable. Finally, the grade of glomerular filtration rate impairment could affect the 24 h urine excretion of urea, overestimating dietary protein intake. For all these considerations, we plan to perform a study estimating protein intake by urea excretion in a cohort of ESKD patients who consume meals with a known protein intake during 24 h urine collection. Finally, to overcome any possible doubt, we plan to include 102 patients in the future study, considering the worst-case scenario for diet adherence derived from a three-day diet diary.

A further limitation of our findings was the duration of this study, which did not allow us to thoroughly assess the long-term effects on disease progression and clinical outcomes. Recognizing this, we are committed to extending the follow-up to over one year to provide more substantial data on this approach’s sustainability and long-term efficacy. Our report focused on Italian patients, who may have peculiar habits and tendencies to follow a low-protein diet. Therefore, evaluating the feasibility of protein counting in other countries is crucial to assessing its value. Future studies would benefit from incorporating new nutritional monitoring technologies, such as a user-friendly app for protein counting, which could enhance diet adherence [58].

### 4.2. Feasibility and Future Study Planning

Finally, our feasibility criteria for the educational support program revealed weaknesses in adherence to the program, considering that the number of meetings and their structure might not be suitable for patients with pressing work commitments or impairments that hinder participation in all meetings. We proposed six sessions for the study patients: four in small groups and two individual meetings with dieticians. Six meetings for working individuals could significantly impact their daily activities or pose challenges for disabled patients to attend. These findings highlight a need to find a way to support better patient attendance in educational programs. Some different strategies can be adopted, such as combining two small-group meetings in one event, planning opportunities to join the small-group meeting online, and modifying the meeting timetable outside of usual working hours. Combining two meetings could reduce attention levels, negatively impacting the ability to internalize significant features. The relationship between attention and memory has long been explored and is likely related to the time and the free time after the presentation [59]. A previous study showed that a one-hour training lesson appears more efficient for learning [60]. Considering the optimal length of the training lesson, we plan to operate the session for no more than one and a half hours in a future study. However, online training modality could also interfere with the learning process. The evidence in other fields was not aligned, showing suboptimal results in e-learning modality or good efficacy [61,62,63]. Finally, the hypothesis of a change in timetables would require further effort from medicine and dieticians, which could not always be possible in all circumstances. In this context, we plan to offer an online educational interactive program for patients who cannot leave their homes or have work constraints that prevent them from attending in-person meetings.

## 5. Conclusions

Adherence to a low-protein diet is mandatory to allow the possible benefits related to the diet regarding kidney disease progression and the metabolic control of chronic disease. Protein counting seems to be an advantageous strategy in managing low-protein intake in CKD patients, as well as improving diet adherence and diet satisfaction. Furthermore, the preliminary results of our pilot study suggest that a more intensive effort should be made to deliver educational efforts, evaluating different strategies to combine face-to-face meetings with interactive online educational meetings to reduce the inconvenience related to difficulties in reaching hospital facilities.

## Figures and Tables

**Table 1:** Clinical characteristics of the patients before and after the educational program.

Variable	T0	T1	*p*
BMI (kg/m^2^)	25.50 (23.3–31.6)	25.8 (3.52–30.27)	0.09 ^b^
eGFR (mL/min/1.73 m^2^)	17 (12.2–22.5)	17 (11.2–22)	052 ^b^
Urea (mmol/L)	18.8 (12.8–23.6)	14.39 (10.9–20.1)	<0.001 ^b^
Sodium (mmol/L)	140 (138–142)	138 (136–142)	0.18 ^b^
Potassium (mmol/L)	4.5 (4.3–4.9)	4.4 (4.1–4.7)	0.17 ^b^
Bicarbonate (mmol/L)	23.3 ± 2.45	24.2 ± 3	0.006 ^a^
Calcium (mmol/L)	2.4 (2.3–2.5)	2.4 (2.3–2.5)	0.385 ^b^
Phosphate (mmol/L)	1.24 ± 0.24	1.18 ± 0.26	0.3 ^a^
PTH (ng/mL)	136 (106–200)	113 (88–176)	0.04 ^b^
25 OH Vitamin D (nmol/L)	72 ± 25.7	90.4 ± 25	0.003 ^a^
Hemoglobin (g/L)	120 (118–128)	114 (109–129)	0.04 ^b^
HbA1c (mmol/mol)	39.75 ± 7.5	39.86 ± 9	0.82 ^a^
Albumin (g/L)	43.6 ± 3.8	41.7 ± 3.7	0.005 ^a^
Total Cholesterol (mmol/L)	4.6 ± 1.3	4.1 ± 1.1	0.03 ^a^
Triglycerides (mmol/L)	1.12 (0.87–1.46)	1.16 (0.84–1.73)	0.93 ^b^
U-Na (mmol/day)	144 ± 69	127 ± 49	0.63 ^a^
U-Urea (mol/day)	0.26 (0.19–0.32)	0.19 (0.15–0.23)	<0.001 ^b^
U-Protein (g/day)	0.65 (0.31–1.75)	0.7 (0.24–1.4)	0.15 ^b^

Footnotes: ^a^ paired *t*-test for normally distributed variables; ^b^ Wilcoxon signed-rank test for non-normally distributed variables; BMI, body mass index; eGFR, estimated glomerular filtration rate; PTH, parathormone; HbA1c, glycated hemoglobin; U-Na, urine sodium excretion; U-Urea, urine urea excretion; U-Protein, proteinuria.

**Table 2:** Diet intake before and after the educational program.

Variable	T0	T1	*p*
Kcal	1663 ± 315	1835 ± 277	0.001
Protein	45.6 ± 9.7	42.3 ± 7.1	0.315
Carbohydrates	206 ± 62.3	247 ± 55	<0.001
Lipids	67 ± 12.3	75.8 ± 11.9	0.02
Fibers	19.1 ± 5.9	24.2 ± 6.6	0.003
Sodium (mg)	2072 ± 685	1877 ± 251	0.27
Potassium (mg)	2141 ± 520	2434 ± 557	0.04
Phosphate (mg)	695 ± 140	681 ± 134	0.5

Footnotes: paired *t*-test for normally distributed variables.

**Table 3:** Levels of satisfaction before and after the educational program.

Variable	T0	T1	*p*
General satisfaction	3.57 (3.1–4)	4 (3.6–4.1)	0.001
Ease in food preparation	4 (3.5–4.4)	4 (4–4.5)	0.11
Social difficulties	2.5 (2.25–3)	2.87 (2.5–3.5)	0.002
Adherence perception	4.66 (4–4.9)	4.8 (4.7–5)	0.001
Motivation	4.25 (3.5–4.5)	4.5 (4.1–5)	<0.001

Footnotes: Wilcoxon signed-rank test for non-normally distribution variables; every item was graded from 1 to 5, where 1 represents total dissatisfaction, and 5 represents complete satisfaction.

## Data Availability

The data presented in this study are available on request from the corresponding authors.

## Abbreviations

The following abbreviations are used in this manuscript:

CKD	Chronic Kidney Disease
eGFR	Estimated Glomerular Filtration Rate
CM	Conservative Management
ESKD	End-Stage Kidney Disease
COPD	Chronic Obstructive Pulmonary Disease
BMI	Body Mass Index
DSQ-MDRD	Dietary Satisfaction Questionnaire- Modification of Diet in Renal Disease
PTH	Parathormone
HbA1c	Glycated Hemoglobin
U-Na	Urine Sodium Excretion
U-Urea	Urine Urea Excretion
U-Protein	Proteinuria

## Appendix A

Satisfactory Diet Questionnaire for Modified Diet in Renal Disease.
  Multiple choice questions.
  Answer scores range from 1 (not satisfied) to 5 (very satisfied).
  General Diet Satisfaction

(a) Are you satisfied with your meal?
  1   2   3   4   5

(b) Do you frequently feel hungry?
  1   2   3   4   5

(c) How would you rate your appetite?
  1   2   3   4   5

(d) Are you satisfied with the flavor of your food?
  1   2   3   4   5

(e) Are you pleased with the amount of food you are eating?
  1   2   3   4   5

(f) Are you happy with the range of food you’re eating?
  1   2   3   4   5

(g) Do you believe that food impacts your health?
  1   2   3   4   5

Ease of preparing meals

(h) Did you find the foods necessary for your diet with ease?
  1   2   3   4   5

(i) Can you effectively organize your meals?
  1   2   3   4   5

Social Challenges Associated with Eating Patterns

(j) How similar do you believe your eating habits are to those of others?
  1   2   3   4   5

(k) Do you think others accept that you have different eating habits than they do?
  1   2   3   4   5

(l) How easy is it to dine at restaurants?
  1   2   3   4   5

(m) How easy is it to eat at someone’s house?
  1   2   3   4   5

Treatment compliance

(n) Did you find the guidance offered by your nephrologist or dietitian helpful?
  1   2   3   4   5

(o) Did you find the program you are following to be helpful?
  1   2   3   4   5

(p) How often do you adhere to the assigned diet (including free meals)?
  1   2   3   4   5

General motivations

(q) Are you satisfied with the foods included in your current diet, or are there foods you enjoy that are not included?
  1   2   3   4   5

(r) How motivated are you to follow the diet?
  1   2   3   4   5
